# Wettability and Interfacial Reaction between the K492M Alloy and an Al_2_O_3_ Shell

**DOI:** 10.3390/ma17184674

**Published:** 2024-09-23

**Authors:** Guangyao Chen, Houjin Liao, Shaowen Deng, Man Zhang, Zheyu Cai, Hui Xu, Enhui Wang, Xinmei Hou, Chonghe Li

**Affiliations:** 1 State Key Laboratory of Advanced Special Steel & Shanghai Key Laboratory of Advanced Ferrometallurgy & School of Materials Science and Engineering, Shanghai University, Shanghai 200444, China; cgybless1@shu.edu.cn (G.C.); hjliao2023@shu.edu.cn (H.L.); dengshaowen@shu.edu.cn (S.D.); manzhang@shu.edu.cn (M.Z.); zycai2022@shu.edu.cn (Z.C.); 2 Shanghai Special Casting Engineering Technology Research Center, Shanghai 201605, China; 3 Institute for Carbon Neutrality, University of Science and Technology Beijing, Beijing 100083, China; d202210667@xs.ustb.edu.cn (H.X.); houxinmeiustb@ustb.edu.cn (X.H.)

**Keywords:** K492M alloy, Al_2_O_3_ shell, interaction, wettability

## Abstract

In this study, wettability behavior and the interaction between the K492M alloy and an Al_2_O_3_ shell were investigated at 1430 °C for 2~5 min. The microstructural characterization of the alloy–shell interface was carried out by optical microscopy (OM) and scanning electron microscopy (SEM). The results indicated that the interaction could cause a sand adhesion phenomenon affecting the alloy, and the attached products were Al_2_O_3_ particles. In addition, the wetting angles of the alloys located on the shell were 125.2°, 109.4°, 97.0°, and 95.0°, respectively, as the contact time was increased from 2 to 5 min. Apparently, the wettability of the alloy in relation to the shell had a relationship with the contact time, where a longer contact time was beneficial to the permeation of the alloy into the shell and the interaction between the two components. No significant chemical products could be detected in the interaction layer, indicating that only the occurrence of the physical dissolution of the shell took place in the alloy melt.

## 1. Introduction

Similar to the IN792 superalloy, the K492M superalloy has been widely used in industrial gas turbines and aero-engine blades because of its medium–high- and high-temperature structural strength and thermal corrosion resistance [1,2,3]. Because of the complex structure of the alloy’s components, investment casting is commonly used to achieve precise control over the dimensions and to prepare the thin wall and lightweight components [4]. In order to obtain a better filling capability for the alloy melt, the alloy melt is brought into contact with a shell in the process of investment casting at a high temperature for a long time [5,6]. However, complex physical and chemical reactions occur between the alloy melt and the shell, leading to surface defects such as sand adhesion. Then, the surface of the alloy requires some machining to eliminate the surface defects, which can influence the dimensional accuracy of the alloy component. Thus, the surface quality and the dimensional stability of alloys have a direct relationship with the interaction in question [7,8,9].

In order to reduce the interaction between the alloy melt and the shell, the selection of a highly stable refractory substrate for the casting shell is very important. To date, Al_2_O_3_ has been considered a relatively suitable refractory substrate for shells during investment casting [10]. Because of the presence of some highly active elements in the alloy melt, the occurrence of an interaction between the alloy melt and the shell is inevitable. Additionally, when the liquid melt comes into contact with the solid shell, the concentration difference in the constituent elements of the shell on the melt side can lead to their diffusion into the melt, resulting in the formation of an adhesion layer on the alloy. Consequently, identifying how to control the interaction between them has become a research focus in order to improve the K492M alloy [11,12,13,14,15]. Currently, the interfacial behavior between the refractory and alloy melts is evaluated by investigating the reactions along their contact interface [16,17,18,19,20,21,22,23]. In addition, contact wettability is a common method used to evaluate the interaction. For example, Li et al. [14] investigated wettability and the interaction between a DZ22B nickel-based superalloy melt and various facecoats. Their results showed that by adding h-BN powder to the fused alumina-based facecoats, the wetting angles between the alloy melt and the facecoats were significantly increased, effectively suppressing the sand-burning defects on the surface of the cast blades. Wang et al. [15] studied the interaction between an Al_2_O_3_-based shell and Y/Y+La-containing single-crystal Ni-based superalloys. Their results indicate that the addition of Y and La simultaneously had the synergetic effect of retarding reactions between reactive REEs and ceramic shells. Chen et al. [23] found that, with increasing C and Hf in a Ni_3_Al-based superalloy, the wetting angles of the alloy melt on ceramic molds decreased and the interface reaction became more severe. Wang examined the wettability and interfacial reactions of Ni-based superalloys using refractory substrates composed of Al_2_O_3_, SiO_2_, ZrSiO_4_, and CoAl_2_O_4_, respectively [24]. The stability of the refractory substrates underwent a systematic evaluation. This approach was used to comprehensively evaluate the interfacial behavior between the refractory and K492M alloy melts and to assess the stability of the proposed Al_2_O_3_ refractory substrate. Notably, there are no research reports related to the aforementioned study.

In this study, the aim was to investigate the interfacial behavior between Al_2_O_3_ shells and the K492M superalloy. The wettability and interaction were studied by measuring the wetting angle and analyzing the interaction product. Based on this, the interaction mechanism between the shell and the alloy melts was also outlined.

## 2. Experiment

In this study, the preparation method for the Al_2_O_3_ shell used a composite approach involving the face layer and back layer. Among the options available, fused Al_2_O_3_ with 325-mesh powder was used as the face layer. Mullite sands with 90-mesh and 54-mesh powders were used as the back layer. To ensure better bonding of the powder, silica sol was selected as the binder. The preparation of the face layer controlled the powder-to-liquid ratio, which was 3.5:1. By adding the dispersants, defoamers, and wetting agents, the slurry obtained better liquidity. After coating the wax with the prepared slurry, a layer of Al_2_O_3_ powder was sprinkled over the surface. The same method was also used to prepare the mullite back layer. Finally, the de-waxing operation was conducted in a de-waxing stove, and the shell was sintered at 950 °C, being held for 4 h in a muffle furnace. The furnace was of a pit-type design, which facilitated the provision of a relatively uniform temperature field with which to calcine the shell.

In this experiment, the K492M superalloy was the raw material. Its nominal composition (wt.%) is shown in Table 1. The alloy was cut into 5 × 5 × 5 mm cubes using wire electrical discharge machining. Before the contact experiment, the oxide layer on the alloy sample was thoroughly polished.

**Figure 1:**
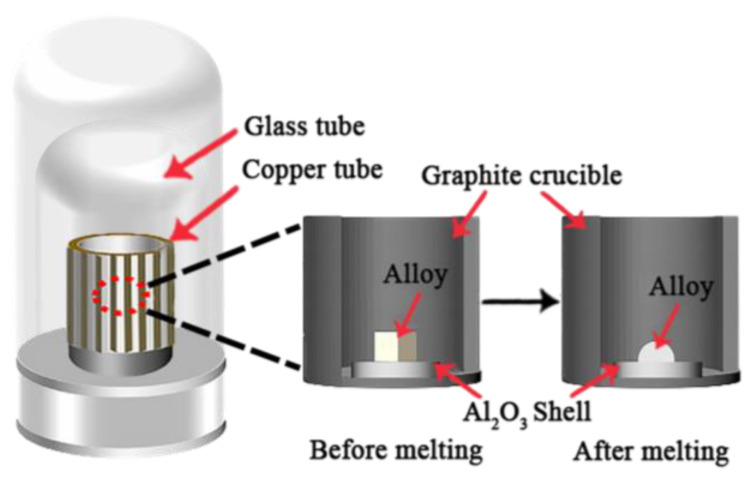
The schematic diagram of the contact experiment apparatus.

The contact experiment was conducted using the non-in situ seating-drop method, as mentioned in our previous study [25], and the experimental setup is shown in Figure 1. In this study, the contact temperature was controlled at 1430 °C and the holding time was set at 2 to 5 min, respectively. After the molten alloy was cooled on the shell to form the hemispherical droplets, the alloy and the shell were taken out for further analysis. The macro- and micro-morphologies of the alloys and shells were examined using optical microscopy (OM) and scanning electron microscopy (SEM, Gemini 300, Oberkochen, Germany), coupled with energy-dispersive spectroscopy (EDS).

## 3. Results and Discussion

### 3.1. Macro-Morphology of the Alloy in Contact with Al_2_O_3_ Shells

**Figure 2:**
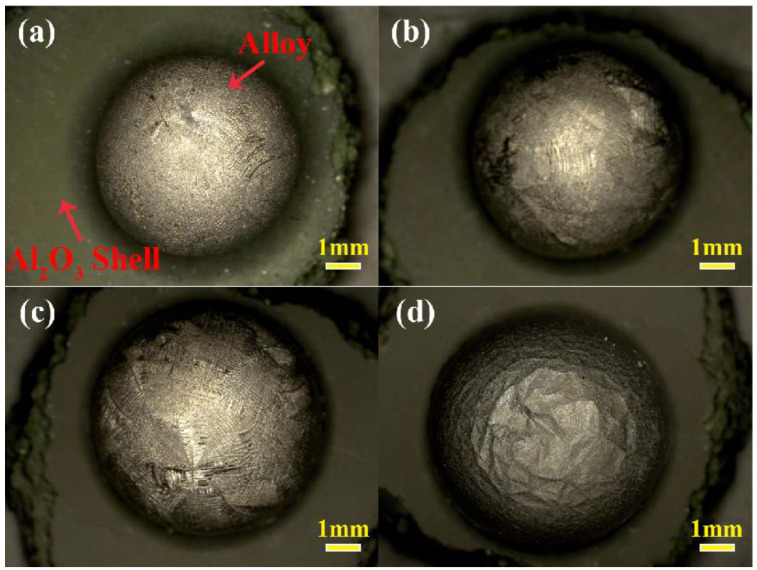
Macroscopic pictures of the top of the alloys located on the Al_2_O_3_ shells after making contact at 1430 °C: (**a**) 2 min; (**b**) 3 min; (**c**) 4 min; (**d**) 5 min.

Figure 2 exhibits macroscopic pictures of contact between the top of the alloys and the Al_2_O_3_ shells. The alloy exhibited a nearly spherical shape after making contact with the shell at 1430 °C for 2 min, as shown in Figure 2a. In addition, the alloy had a significant metallic luster, indicating that no oxidation occurred on the surface of the alloy and that it was not contaminated by the surroundings. From Figure 2b–d, it can be seen that as the contact time was increased from 3 to 5 min, no significant changes occurred in the macroscopic appearance of the alloys.

**Figure 3:**
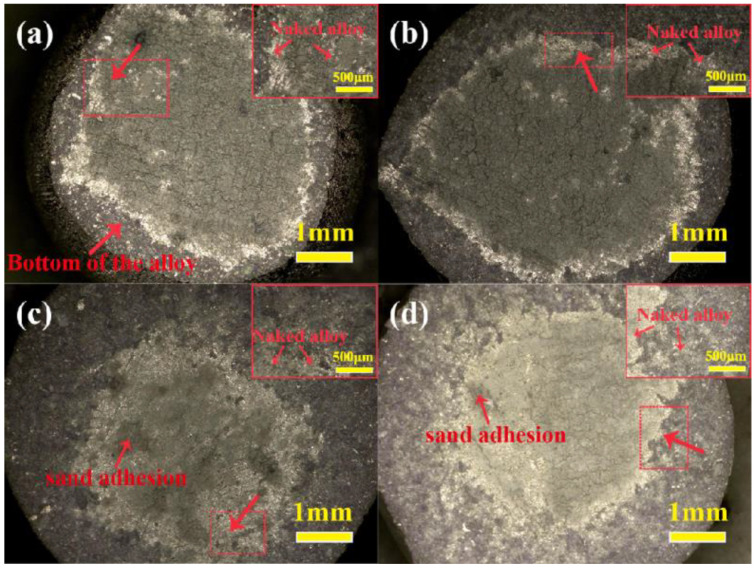
Macro-morphology at the bottom of the alloys in contact with the Al_2_O_3_ shells at 1430 °C: (**a**) 2 min; (**b**) 3 min; (**c**) 4 min; (**d**) 5 min. The insets in the upper right corner of Figure 3a–d correspond to enlarged views of the respective dashed-frame areas.

In order to analyze the sand adhesion of the alloy, the macro-morphology of the bottom of the alloys in Figure 2 is shown in Figure 3. It can be seen that an obvious gray substance (referred to as sand adhesion) was observed at the bottom of the alloy in contact with the shell at 1430 °C for 2 min, as shown in Figure 2a. In addition, some silvery-white areas were exposed at the bottom of the alloy; it can be confirmed that it was the naked alloy. From Figure 3b–d, it can be seen that a similar sand adhesion phenomenon also appeared at the bottom of the alloys in contact with the alloy melts for 3~5 min. However, the exposed area of the naked alloy exhibited a decreasing trend as the contact time increased. Apparently, the appearance of the sand adhesion was caused by the interaction between the alloy and the shell. Chen’s [26] study showed that the extent of the sand adhesion had a direct relationship with the extent of interaction. Thus, it could be concluded that the extent of interaction in this study was further intensified as the contact time increased.

**Figure 4:**
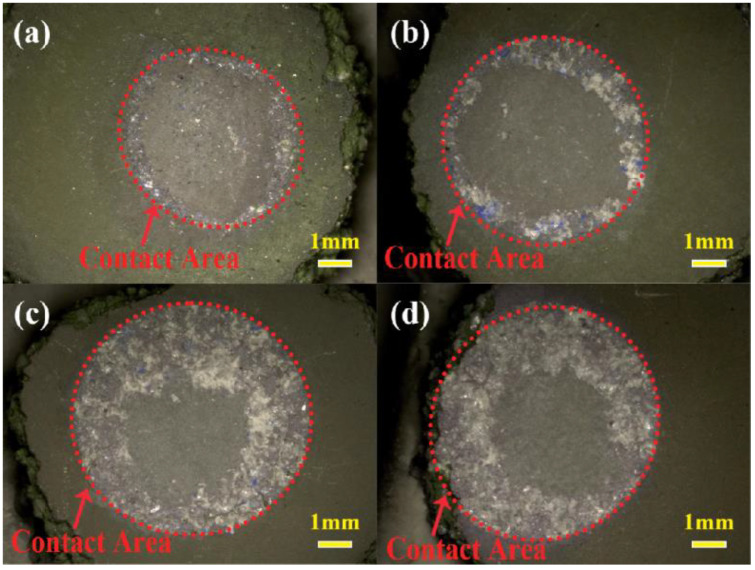
Macro-morphology of the surface of the shells in contact with the alloys at 1430 °C: (**a**) 2 min; (**b**) 3 min; (**c**) 4 min; (**d**) 5 min.

Figure 4 shows macroscopic pictures of the surface of Al_2_O_3_ shells in contact with the alloys at 1430 °C for 2~5 min, respectively. In Figure 4a, the contact area of the shell appears nearly circular in shape. This is because the alloy melt tends to form a spherical shape due to the surface tension, resulting in a circular contact surface with the shell. There is a noticeable separating phenomenon on the surface of the shell at the edges in contact with the alloy. In comparison with Figure 4a, a similar phenomenon is observed in Figure 4b; however, the separating area exhibits an increasing trend. In addition, a more serious phenomenon can be observed in Figure 4c,d after 4~5 min of contact. This indicates that the interaction between the alloy and the shell intensified.

### 3.2. Microscopic Morphology of Al_2_O_3_ Shells in Contact with the Alloy

**Figure 5:**
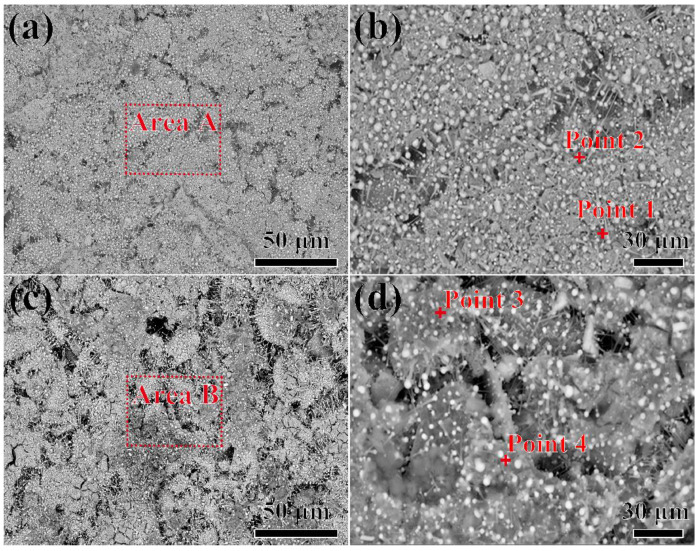
SEM pictures of the surface of Al_2_O_3_ shells in contact with the alloys at 1430 °C for 2~3 min, respectively: (**a**) shows the results after 2 min; (**b**) is a magnified picture of area A in (**a**); (**c**) shows the results after 3 min; (**d**) is a magnified picture of area B in (**c**).

The microstructure of the surface of Al_2_O_3_ shells after contact with the alloys at 1430 °C for 2~3 min is shown in Figure 5. In Figure 5a, it can be seen that many small particles are observed on the surface of the shell and they are uniformly distributed. A magnified picture of area A (in Figure 5a) is depicted in Figure 5b. In combination with the analysis of point 1 (in Table 2), it could be confirmed that it was the Al_2_O_3_ shell. In addition, point 2 is composed of Al, Ni, Cr and Co elements, indicating that the particles are the residual alloy. The residual alloy only adhered to the surface of the shell without the interpenetration phenomenon. Furthermore, the residual alloy particles exhibited spherical and needle-like shapes, respectively. When the molten alloy shrank on the surface of the shell, the alloy melt remained on the surface of the shell due to its wettability with the shell. Figure 5c shows a phenomenon similar to that in Figure 5a. A magnified picture of area B (in Figure 5c) is shown in Figure 5d. The analysis of point 3 and 4 (shown in Table 2) exhibited that only the Al_2_O_3_ matrix and the attached alloy were detected. No other new products were observed. It could be concluded that only physical interactions occurred between the shell and the alloy melt. Additionally, in comparison with the surface of the shell with a contact time of 2 min, the structure of the surface of the shell with a contact time of 3 min was looser, with an increased number of pores and larger pore sizes. During the contact process between the alloy melt and the shell, a certain degree of interaction occurred, leading to mass transfer. The amount of element diffusion was related to the contact time. Additionally, due to varying amounts of sand adhesion to the bottom of the alloy over extended periods, the surface of the shell exhibited noticeable differences at different times.

**Figure 6:**
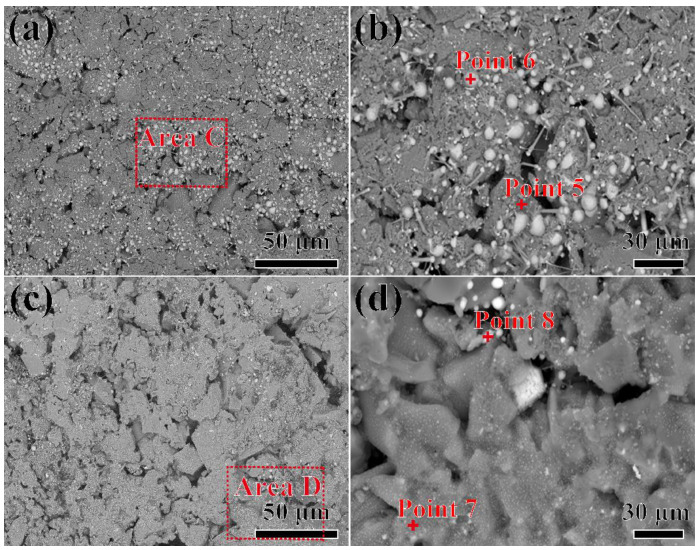
SEM pictures of the surface of Al_2_O_3_ shells in contact with the alloys at 1430 °C for 4~5 min, respectively: (**a**) shows the results after 4 min; (**b**) is a magnified picture of area C in (**a**); (**c**) shows the results after 5 min; (**d**) is a magnified picture of area D in (**c**).

The microstructure of the surface of the shell after contact with the alloy melt at 1430 °C for 4~5 min is shown in Figure 6. In Figure 6a, the gray particles on the surface of the shell exhibit a lower content than those shown in Figure 5a. A magnified picture of area C (in Figure 6a) is shown in Figure 6b. In Figure 6b, it can be seen that the small spherical particles disappeared and only large-sized spherical particles remained on the shell. The EDS results in Table 3 indicated that points 5 and 6 corresponded to the Al_2_O_3_ shell matrix and the residual alloy, respectively. After the alloy melt made contact with the shell for 5 min, the amount of the residual alloy exhibited a further decreasing trend, as shown in Figure 6c. Basically, no large-sized spherical alloy particles appeared on the surface of the shell. The magnified picture of area D in Figure 6c is shown in Figure 6d. In Figure 6d, the Al_2_O_3_ shell matrix (point 7) is clearly visible, and only a small number of alloys (point 8) are visible. It is evident that the interaction between the alloy and the shell can affect their wettability. A longer contact time meant that the alloy could have deeper penetration into the shell, leading to a smaller amount of the alloy remaining on the surface of the shell after separation.

**Figure 7:**
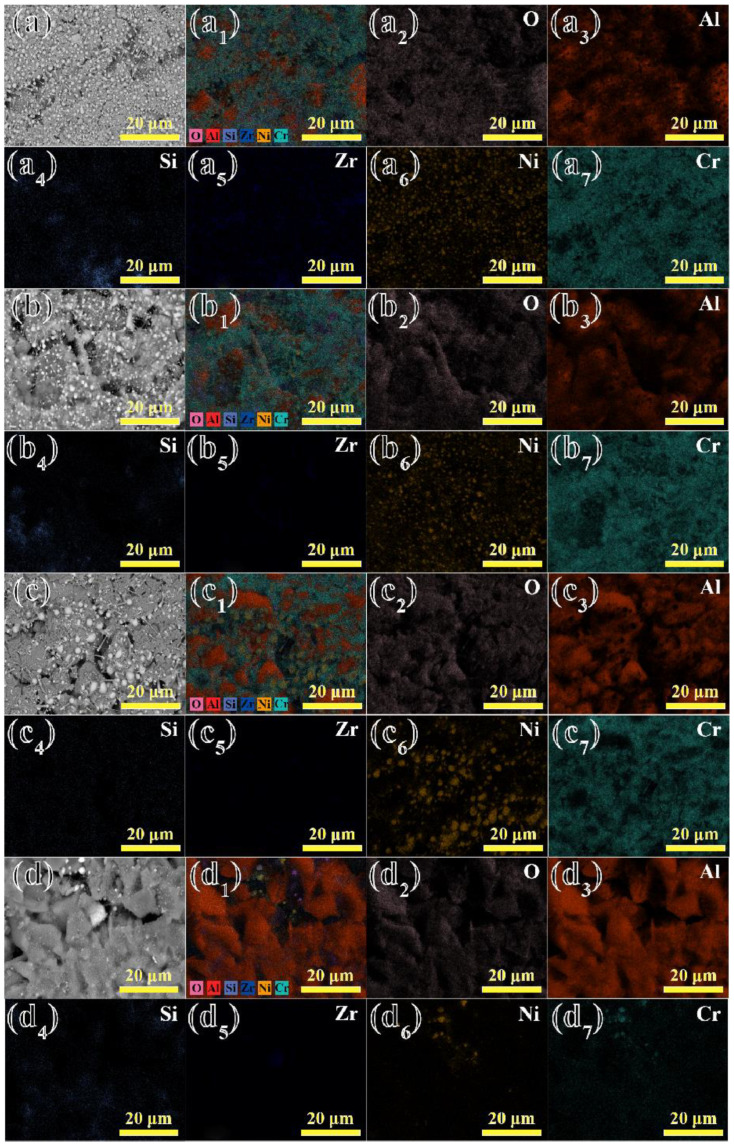
EDS elemental mapping pictures of the surface of Al_2_O_3_ shells in contact with the alloys at 1430 °C for 2~5 min, respectively: (**a**–**a_7_**) 2 min; (**b**–**b_7_**) 3 min; (**c**–**c_7_**) 4 min; (**d**–**d_7_**) 5 min.

The EDS elemental mapping pictures for the surface of the shell after contact with the alloys at 1430 °C for 2~5 min are shown in Figure 7. In Figure 7a–a_7_,b–b_7_, it is shown that the surface of Al_2_O_3_ shell mainly consisted of Al and O elements, along with Si and Zr elements. The residual alloys were mainly composed of Al, Ni, Cr and other elements, which is consistent with the analysis presented in Figure 5. In Figure 7a–d, it can be seen that the residual Ni elements exhibited a significant downward trend. Thus, the increase in contact time between the alloy and the shell could enhance their wettability. In addition, it can be seen that many Cr elements were distributed across the surface of the shell. Consequently, the surface of the shell might appear somewhat green, which is consistent with the phenomenon shown in Figure 4. Liu et al. show that the relative stability of the oxides, from high to low, was as follows: Al_2_O_3_ > TiO_2_ > Cr_2_O_3_ > CoO > NiO [27]. In addition, due to the low oxygen content from diffusion, it was difficult to detect the presence of TiO_2_ residues. However, the Cr element also had a strong affinity for the O element, and so it tended to remain on the surface of the shell in substantial quantities along with the alloy melt.

### 3.3. Microstructure of the Cross-Section of the Alloys in Contact with the Shells

**Figure 8:**
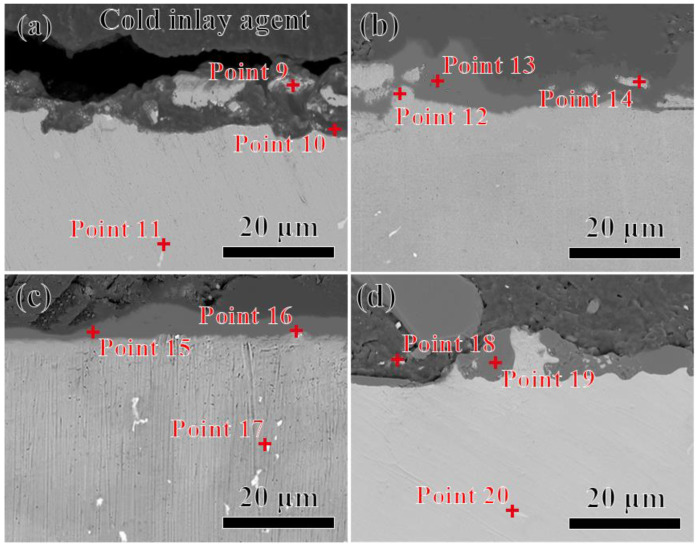
SEM pictures of the cross-section of the alloys in contact with Al_2_O_3_ shells at 1430 °C for 2~5 min, respectively: (**a**) 2 min; (**b**) 3 min; (**c**) 4 min; (**d**) 5 min.

The microstructure of the cross-section of the alloy in contact with the Al_2_O_3_ shell at 1430 °C for 2~5 min is shown in Figure 8. Figure 8a shows that a 10 μm thick black layer (interaction layer) was attached to the gray-white alloy layer after being in contact with the shell for 2 min. The EDS results of points 9~11 in Table 4 indicate that the interaction layer is a mixture of the Al_2_O_3_ shell and the alloy, and that the gray-white is the alloy matrix. Apparently, during the contact experiment, the molten alloy could infiltrate into the shell, and the rapid shrinkage of the alloy melt could cause the generation of a mixture during the rapid cooling process. Figure 8b shows the cross-section of the alloy after contact with the shell for 3 min. A similar phenomenon is observed as Figure 8a. An adhesion layer with a thickness of 15 μm is found along the alloy matrix. The EDS results of points 12~14 in Table 4 indicate that the alloy matrix and adhesion layer were distinguishable, respectively. In addition, the adhesion layer also consisted of Al_2_O_3_ and the alloy, as indicated in point 13. From Figure 8c,d, it can be seen that as the contact time was increased from 4~5 min, no significant changes occurred between the interaction layer and the alloy matrix. After 5 min of contact, there were large-sized Al_2_O_3_ particles attached to the alloy matrix. This indicates that the extension of the contact time increases the penetration of the alloy melt into the interior of the shell. A study by Long showed that the chemical activity of the Ni-based superalloys was relatively low, indicating that no chemical reaction would occur between the alloy melt and the Al_2_O_3_ shell [28]. In addition, no chemical products were observed in Figure 8, indicating that only the physical dissolution of Al_2_O_3_ shell occurred in the alloy melt, which is consisted with the analysis in Figure 7.

**Figure 9:**
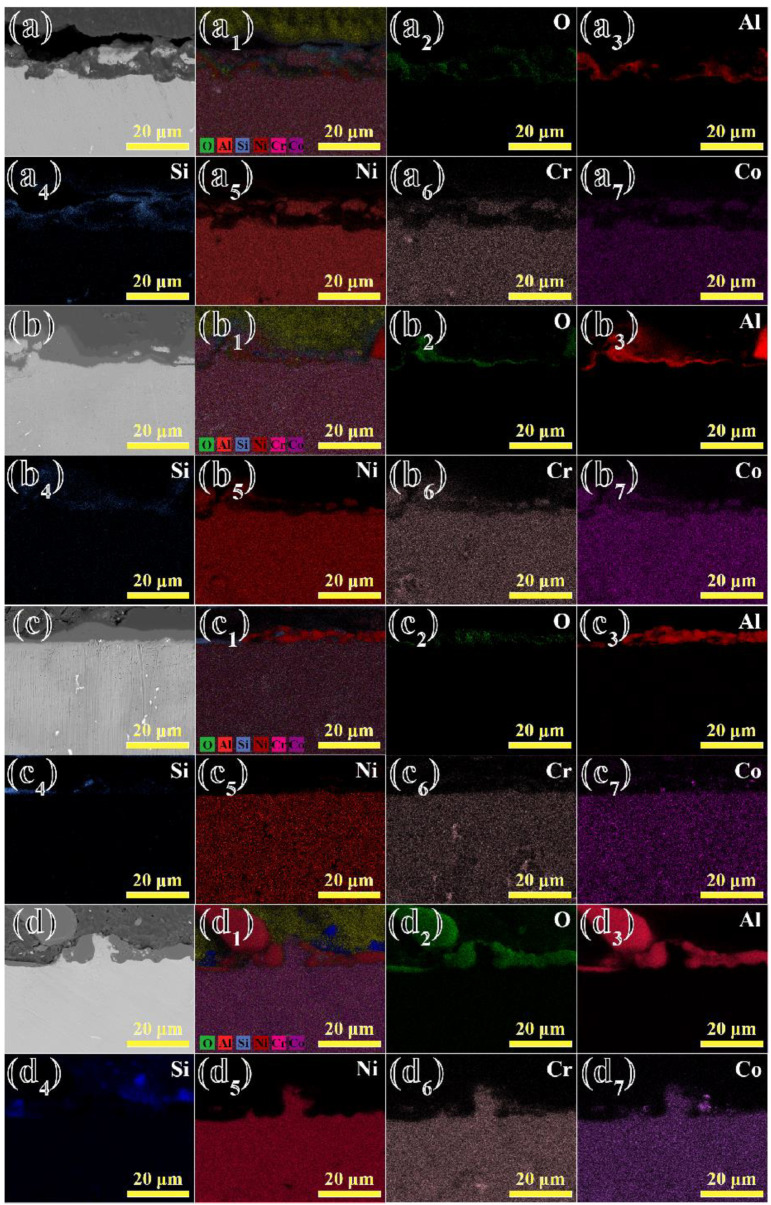
EDS elemental mapping pictures of the cross-section of the alloys in contact with Al_2_O_3_ shells at 1430 °C for 2~5 min, respectively: (**a**–**a_7_**) 2 min; (**b**–**b_7_**) 3 min; (**c**–**c_7_**) 4 min; (**d**–**d_7_**) 5 min.

Figure 9 shows the microstructure of the longitudinal cross-section of the contact layer in the alloy prepared at 1430 °C for 2~5 min. In Figure 9a–a_7_,b–b_7_, it is shown that the interior of the alloy mainly consisted of Ni, Cr, and Co elements. In addition, the same phenomenon is shown in Figure 9c,d. The alloy and Al_2_O_3_ shells underwent physical dissolution, forming an interaction layer of a certain thickness, in which large amounts of Al and O elements were enriched. In fact, researchers had reported that the interaction between nickel-based superalloys and Al_2_O_3_ shells was mainly controlled by physical dissolution diffusion [29]. Alloys that reacted with the shell for different durations formed a reaction layer, which was predominantly composed of Al and O elements. Although small amounts of Ni elements were dispersed in the reaction layer, they did not participate in the interaction to form NiO. It is speculated that the regions where Ni elements were detected corresponded to the areas of the alloy matrix that had not yet reacted with the shell. In addition, some large-sized Al_2_O_3_ particles could be observed adhering to the alloy side as the contact time increased. This might be due to longer contact time leading to an increasing trend of sand adhesion, resulting in large particles attaching more easily to the alloy side.

### 3.4. Wetting Behavior between the Alloy and the Shells

**Figure 10:**
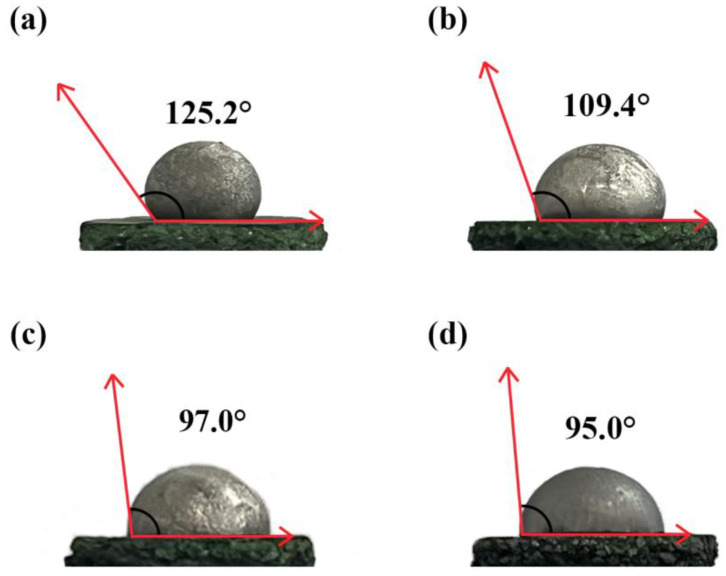
The wettability angle pictures of the alloy located on the Al_2_O_3_ shells after contact at 1430 °C: (**a**) 2 min; (**b**) 3 min; (**c**) 4 min; (**d**) 5 min.

Figure 10 shows the front view of the macro-morphology of the alloy in contact with the shell at 1430 °C. Apparently, all of the alloys had a spherical crown shape after contact with the shell. This occurred because, when the block shape alloy was melted, it initially took a spherical shape due to surface tension. Subsequently, during contact with the shell, the interaction between the alloy and the shell could influence the wetting state of the alloy. In addition, the alloys had a smooth and clean surface, indicating that the alloy was not seriously contaminated by the surroundings in the furnace. As the contact time was increased from 2 to 5 min, the alloy drops’ bottoms became narrower, as shown in Figure 10a–d. The measured angles of the alloys were 125.2° (Figure 10a), 109.4° (Figure 10b), 97° (Figure 10c), and 95° (Figure 10d), respectively. The results show that an increase in contact time leads to a gradual decrease in the wetting angle of the system. In addition, it was confirmed that the wetting angle exhibited a decreasing trend as the interaction extent increased. The K492M alloy is widely used for the blades of industrial gas turbines and aero-engines and in integral turbines in auxiliary power units (APUs). In addition, these components are primarily prepared using investment casting. During this process, the molten alloy entered into the Al_2_O_3_ Shell, and the wettability of the alloy melt influenced its filling capability as well as the surface quality of the alloy casting. This study investigated wettability and the interaction between the K492M alloy and the Al_2_O_3_ shell, providing theoretical guidance for the investment casting of the alloy in the future.

## 4. Conclusions

In this study, the interaction behavior between the K492M superalloy and the Al_2_O_3_ shells was investigated at 1430 °C for 2~5 min, respectively. The conclusions are summarized as follows:

(1) After the contact experiment, the interaction between the alloy and the shell caused sand adhesion, and the extent of the sand adhesion increased with a longer contact time.
(2) With the extension of contact time, significant interactions occurred between the shells and the alloy, resulting in a thicker interaction layer and more pronounced delamination. This interaction layer was a mixture of Al_2_O_3_ particles and the alloy.
(3) After 2~5 min of contact, the wetting angles of the alloys located on the shells were 125.2°, 109.4°, 97° and 95°, respectively. Obviously, the wettability of the alloys on the shells was related to the contact time. Prolonging the contact time was beneficial for the interaction and significantly improved wettability.
(4) Generally, the wettability of the K492M alloy melt on the shell depended on the contact temperature and time. To improve the surface quality and mold-filling ability of the alloy castings, it was necessary to reduce interactions while controlling wettability to a certain level. Further research was needed to study the changes in wettability at different temperatures and times, and to evaluate and control the actual parameters in combination with the performance of practical castings.

## Figures and Tables

**Table 1:** The nominal element composition of the K492M superalloy.

Alloy	Element/wt.%										
	Co	Cr	W	Al	Ta	Ti	Mo	C	B	Zr	Ni
K492M	9.0	12.5	4.0	3.4	4.1	4.1	2.0	0.09	0.015	0.11	Bal.

**Table 2:** EDS results of points 1~4.

Point	Element/at%						
	O	Al	Si	Zr	Ni	Cr	Co
1	57.98	22.33	1.15	0.84	6.83	8.33	2.54
2	16.35	18.71	2.05	/	41.87	15.34	5.68
3	55.80	39.93	0.43	0.26	0.24	3.17	0.18
4	13.25	18.71	1.38	/	45.21	16.24	5.21

**Table 3:** EDS results of points 5~8.

Point	Element/at%						
	O	Al	Si	Zr	Ni	Cr	Co
5	64.29	30.25	0.52	0.30	0.95	3.37	0.31
6	11.21	26.69	0.46	0.82	51.98	7.15	1.69
7	50.17	41.08	1.59	0.54	3.93	1.21	1.49
8	19.31	43.17	0.10	1.39	20.20	7.31	8.51

**Table 4:** EDS results of points 9~20.

Point	Element/at%						
	O	Al	Si	Ni	Cr	Co	W, Ta, Ti, Mo, Zr
9	/	5.17	/	24.53	5.15	3.32	Bal.
10	18.48	6.54	/	14.72	3.74	2.04	Bal.
11	/	1.53	/	22.91	6.39	3.18	Bal.
12	/	3.35	/	27.83	6.92	4.11	Bal.
13	46.29	35.58	2.56	10.30	2.69	1.76	Bal.
14	6.49	3.36	4.49	21.56	4.13	2.92	Bal.
15	15.39	5.31	9.33	25.18	4.94	3.53	Bal.
16	/	4.50	7.20	24.04	4.95	3.47	Bal.
17	/	5.75	/	32.15	11.48	7.06	Bal.
18	8.93	11.41	3.62	22.35	2.60	2.67	Bal.
19	48.99	37.72	2.28	7.19	2.07	1.98	Bal.
20	/	3.12	/	34.87	12.61	6.55	Bal.

## Data Availability

The original contributions presented in the study are included in the article, further inquiries can be directed to the corresponding authors.
